# Enhancing Resilience in Biometric Research: Generation of 3D Synthetic Face Data Using Advanced 3D Character Creation Techniques from High-Fidelity Video Games and Animation

**DOI:** 10.3390/s24092750

**Published:** 2024-04-25

**Authors:** Florian Erwin Blümel, Mathias Schulz, Ralph Breithaupt, Norbert Jung, Robert Lange

**Affiliations:** 1 Institute for Safety and Security Research, Bonn-Rhein-Sieg University of Applied Science, Grantham-Allee 20, 53757 Sankt Augustin, Germany; florian.bluemel@h-brs.de (F.E.B.); norbert.jung@h-brs.de (N.J.); robert.lange@h-brs.de (R.L.); 2 Federal Office for Information Security, Heinemannstraße 11-13, 53175 Bonn, Germany; ralph.breithaupt@bsi.bund.de

**Keywords:** synthetic biometric data, face biometrics, face data, avatars, realism, resilience, diversity

## Abstract

Biometric authentication plays a vital role in various everyday applications with increasing demands for reliability and security. However, the use of real biometric data for research raises privacy concerns and data scarcity issues. A promising approach using synthetic biometric data to address the resulting unbalanced representation and bias, as well as the limited availability of diverse datasets for the development and evaluation of biometric systems, has emerged. Methods for a parameterized generation of highly realistic synthetic data are emerging and the necessary quality metrics to prove that synthetic data can compare to real data are open research tasks. The generation of 3D synthetic face data using game engines’ capabilities of generating varied realistic virtual characters is explored as a possible alternative for generating synthetic face data while maintaining reproducibility and ground truth, as opposed to other creation methods. While synthetic data offer several benefits, including improved resilience against data privacy concerns, the limitations and challenges associated with their usage are addressed. Our work shows concurrent behavior in comparing semi-synthetic data as a digital representation of a real identity with their real datasets. Despite slight asymmetrical performance in comparison with a larger database of real samples, a promising performance in face data authentication is shown, which lays the foundation for further investigations with digital avatars and the creation and analysis of fully synthetic data. Future directions for improving synthetic biometric data generation and their impact on advancing biometrics research are discussed.

## 1. Introduction

In biometric research, the availability of biometric data that is representative for a certain use case and of high quality is an important factor for both development (training) and a comprehensive evaluation of biometric systems [1]. The acquisition process of these data is often limited by either cost, time, or legal factors (in countries with strong GDPR laws) [2,3,4] and the resulting unbalanced collections of real human attributes can lead to unwanted bias, preventing a trained algorithm from operating in a fair and accurate manner [5]. Another aspect that limits current biometric research is the missing ground truth information in real data, as well as the use of empirical metrics without a thorough understanding of the underlying parameters that influence their calculation and interpretation. The lack of ground truth in biometric research and development even amplifies the need for more data and generally limits the significance of all statistical analysis results. Even the most basic performance metrics FAR and FRR are always based just on the assumption that the database in use is representative and scalable to all intended application scenarios. For the evaluation and certification of biometric applications with high security requirements, these issues become increasingly impractical.

A promising alternative when facing these challenges is the use of synthetically generated data. Although biometrics is a broader term describing various methods of identifying individuals based on their unique characteristics, we focus specifically on facial biometrical data as a subset. Synthetic data can be generated in many ways, each of which has advantages and disadvantages. Those methods will be discussed in Section 2 as part of the related work. Synthetic data aim to reduce the costs of data collection processes by providing unsupervised generation of data in usually unproductive times or via on-demand generation, allowing access to synthetic data without being constrained by the availability of real data. With full control over the generation process, researchers can specify the characteristics and attributes of their desired data, providing ground truth information, which is usually unknown in real data. By systematically varying parameters during data generation and observing their effects, this can lead to a deeper understanding of parameters’ influence on the performance metrics of biometric systems, which could affect the system design and the optimization of those systems. The objective of this work is to assess facial images of synthetic characters generated by 3D graphic tools, particularly utilizing character creation software tailored for movies and games, in relation to their adequacy as a substitute for real data through an equivalence approach. Our findings contribute to the field of biometric research and implicate advancements in data acquisition processes and available ground truth information in data. Section 3 covers the mentioned generation of the characters and the tools used. Acquisition of reference data and further insights into the comparison between rendered synthetic face images and real images can be found in Section 4. In Section 5, we address the limitations and challenges our method encounters. Finally, we reflect on our results and discuss future directions and prospects. With fully synthetic characters, the privacy of individuals is not a concern; 3D-generated synthetic characters can provide ground truth information that enhances the understanding of influencing parameters while providing arbitrarily large datasets. 

## 2. Related Work

Generation of synthetic data has become a staple of biometric research over the past couple of years. A method that has recently attracted a significant amount of interest and has been utilized for generating such synthetic biometric data involves the use of deep neural networks (DNNs) [6], including convolutional neural networks (CNNs) and recurrent neural networks (RNNs), by creating face images through pre-trained models. These networks can capture complex patterns and dependencies in the data, enabling the generation of realistic and diverse synthetic samples. A problem with the use of DNNs is over-optimization due to too-small datasets, which could result in information leaks from training data [2,7], thus failing to comply with data protection laws and limiting the capabilities of DNNs. Due to general explainability issues in neural networks, it is never clear how accurate and representative the results are. The problem of using an insufficient amount of training data is also a consideration when using character creation tools; however, this method offers the advantage of precisely selecting and deselecting characteristics and their parameters to ensure that training data are not revealed in the generation process. This possibility allows for greater accuracy when compared with DNNs or the next method presented. 

Generative Adversarial Networks (GANs), like StyleGAN, have been widely used for generating synthetic biometric data [8,9,10]. They consist of a generator network, which produces synthetic data, and a discriminator network, which tries to distinguish between real and synthetic data. Through an adversarial training process, GANs learn to generate realistic synthetic samples that closely resemble the distribution of real data. Although GANs currently produce higher-fidelity face images than character creation tools, their limited ability to generate accurate faces from different perspectives impacts their generalizability in use cases negatively. Another issue with GANs is their limited parameterizability. Several limitations are effectively addressed with the sophisticated customizability of the characters’ visual appearance through character creation tools. Animation systems and rigging methods with inverse kinematics and blendshapes enable precise control over the pose in the 3D space and allow for realistic facial expressions. 

Other methods used for synthesis, such as data augmentation, parametric modelling, or input perturbation, add additional data based on real datasets. Real data are augmented by generating additional synthetic samples with variations in pose, lighting conditions, expressions, or other relevant factors. This augmentation enriches the dataset, enhances the diversity of the training data, and improves the robustness of the biometric systems [2,11,12]. While data augmentation offers high fidelity as well, it lacks appropriate compliance with data protection laws.

Techniques for the domain adaptation method aim to bridge the gap between synthetic and real data domains. These approaches focus on minimizing the differences between synthetic and real data distributions, enabling better generalization of biometric systems trained on synthetic data to real-world scenarios [2]. Despite offering better generalization, the techniques and methods mentioned before do not aim to create ground truth data, which are especially of value in biometric research and in the training of biometric authentication systems.

First approaches allowing the conditional generation of synthetic biometrics with specific characteristics or attributes such as age, gender, ethnicity, or pose variations have been explored [13]. Most current technologies in the field of synthetic data for biometric applications depend on the use of neural networks or variation in existing data. While those processes often provide high realism and quality, training data are prone to leaks, and the quality of the network and the resulting quality and diversity are dependent on the availability of high-quality training data [14]. Acquiring such training data is often difficult due to a multitude of reasons.

Privacy concerns and the accompanying laws (e.g., GDPR) make collecting and storing biometric data difficult, as they are rated as highly personal and therefore need to be protected, which makes storing these data a non-trivial task in terms of data security [15]. While scraping the web for training data would be a simple task, it would violate the common data protection laws of many countries. Having subjects provide the data ‘themselves’ leads to another difficulty: capturing the respective data with sufficiently high quality requires equipment and expertise and is time- and cost-intensive. This limits the number of data and their diversity drastically. The demographic of subjects will most likely represent that of the area surrounding the institution. Networks trained with such limited datasets will have a certain bias [2].

## 3. Generating Synthetic Characters

As seen in previous work, synthetic data creation has gained growing attention [2,12]. The tools currently on the market that achieve the desired level of realism for fully synthetic data are sparse, but two of them have emerged as promising solutions for generating realistic synthetic 3D characters, MetaHuman Creator [16] and Character Creator 4. We examined the advantages and disadvantages, and capabilities and limitations of both tools for specific needs. 

### 3.1. Digital Avatars—Semi-Synthetic Data

A subset of synthetic data, which is needed to evaluate the realism of created synthetic data when compared with real data, comprises synthesized authentic data. With a digital avatar, a digital representation of the human characteristics of a reference person, the behavior of face recognition algorithms with both the reference data and their respective digital synthetic data can be analyzed. Digital avatars can closely resemble the physical appearance of real individuals, capturing their facial features, expressions, and body movements and placing them in a controlled and standardized virtual environment, thus eliminating external variables and ensuring consistent data. Therefore, digital avatars provide a link between real data and fully synthetic data, which is crucial to analyzing their performance behavior for biometric authentication.

As for the 3D render engines, MetaHuman Creator v2.0.4 with its Mesh2MetaHuman feature integrated in the MetaHuman Unreal Engine toolkit v3.0.4 can be used to create such digital avatars. A 3D mesh is used as a reference to generate the complete character. The mesh is generated using a multi-camera system, K13, in which 13 cameras are aligned in a half-dome to simultaneously capture photos of a person from 13 defined angles. These are aligned using photogrammetry to generate the 3D mesh. Performance scores matching faces with each other range between 0 and 1 and are calculated using state-of-the-art, market-leading face recognition technologies. A score of 0 refers to no similarity, while a score of 1 refers to the probe and candidate image being identical. A typical score of different images of the same person usually results in a score above 0.9. 

**Figure 1:**
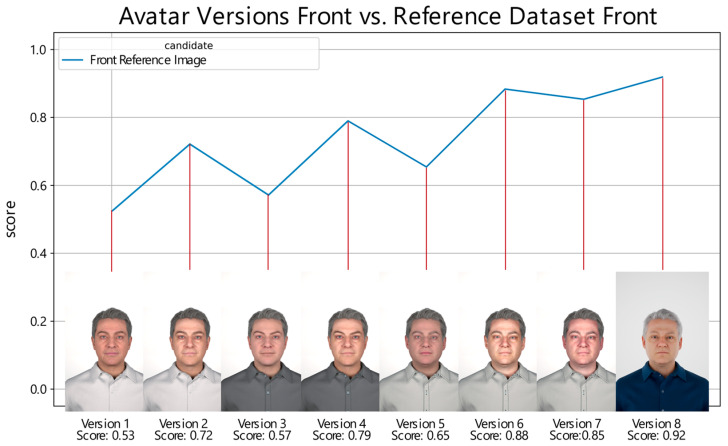
Performance scores of different digital avatar versions when compared with their respective real reference image. The *x*-axis represents the versions of the digital avatars. For each version, an image of the corresponding digital avatar is displayed for visual reference. The *y*-axis denotes the score. The score obtained by each digital avatar version is labeled accordingly beneath the images.

Scores obtained by earlier and current digital avatar versions when compared with the reference image of an individual are visualized in Figure 1. The first generations of digital avatars lacked visual realism, leading to performance scores of less than 0.8, which can be improved through custom-generated maps. Observable regressions in the score of versions one, three, and five occurred when a new mesh with a default texture was generated and compared. The custom-generated maps apparent in versions two, four, and six to eight always tend to increase scores relative to their default textured versions. The texture map represents the color information of the reference data. Normal maps refer to bump maps, which calculate how lighting affects detailed 3D structures, and cavity maps calculate how lighting affects small high-frequency details. These are implemented in MetaHuman’s standard human skin material, which offers parameterization of skin properties. Digital avatars created using MetaHuman Creator and placed in Unreal Engine allow for animation of the character by either applying a prerecorded animation, or through real-time motion capture. Current limitations in customizability options, such as the limited availability of hair and texture options, restrict the ability to achieve highly realistic looks in digital representations.

### 3.2. Fully Synthetic Characters

**Figure 2:**
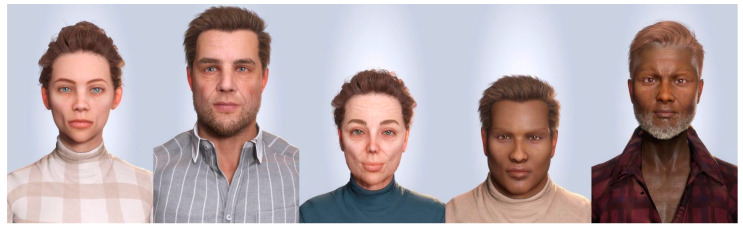
Frontal view of five fully synthetic 3D characters generated with Reallusion’s Characters Creator v4.4.2509.1 at the Biometric Evaluation Center (BEZ). The characters show different heights, ethnicities, skin textures, and ages as well as standardized lighting, background, and camera settings.

When it comes to generating data for biometric research, there are three main options: fully synthetic data (as seen in Figure 2), semi-synthetic data (as seen in Figure 1), and real data. Fully synthetic data offer several benefits to datasets including real data. Fully synthetic biometric datasets provide complete control and customization over the dataset generation process. The desired distribution, variability, and characteristics of the biometric traits can be designed to suit the requirements of the research questions. This control allows for targeted research and facilitates the exploration of specific scenarios, potential vulnerabilities, and edge cases that may be challenging to obtain with real or semi-synthetic data [17]. The generation of 3D synthetic data extends the customization further, allowing for different camera angles, lighting scenarios, objects, and human interactions for the exact same individuals.

Real biometric data are sensitive and subject to privacy concerns. Generating fully synthetic biometric datasets helps overcome these privacy challenges. By using synthetic data in research, the risks associated with handling and storing personal information can be eliminated, reducing the likelihood of data breaches or misuse. This enables more ethically sound research while complying with privacy regulations and safeguarding individuals’ sensitive information, and huge, standardized databases can be shared. Synthetic datasets can easily be scaled up to accommodate large sample sizes, allowing the evaluation of the performance of biometric systems in various scenarios. Additionally, the generation process can be reproduced, ensuring consistency across experiments, and enabling fair comparison between different algorithms or techniques. Biometric systems can be susceptible to bias, leading to inaccuracies and discrimination in identification or authentication processes. By generating fully synthetic datasets for research, data that are free from the inherent biases present in real datasets can be used. These datasets also allow for the intentional introduction of controlled biases, enabling bias-related challenges to be researched and addressed systematically.

Of the two explored methods of generating a large dataset of realistic 3D characters, Reallusion’s Character Creator has proven to be the more viable option for our purposes. The software was designed for generating characters for video games and animation. Its feature set includes many of the benefits mentioned before and allows high customization, such as variable face and body geometry, high-fidelity skin textures and transparency, lighting, backgrounds, and clothing. Changes in facial structure are achieved via several hundred interacting parameters relating to specific facial features. These parameters create an inherent ground truth and detailed annotation for the generated characteristics, making them ideal for neural network training data. Additionally, the vertex numeration of different characters is the same, making the same vertices correspond to the same positions for facial landmarks. Bulk generation of synthetic characters using MetaHuman Creator is very limited due to the user interface and lack of public APIs. Both options prohibit the training of neural networks using data generated with the respective software. A third option without this limitation is currently being explored. 

## 4. Face Recognition Analysis

### 4.1. Reference Data

To evaluate synthetic biometric data, comparison between real and synthetic biometric data is vital. While these data are scarce and hard to come by when considering data privacy and security, the authors at the BEZ of the H-BRS have the opportunity to collaborate with students, coworkers, and external parties, who have agreed to participate in our long-term evaluation process. The evaluation includes capturing multiple sets of reference data for all test subjects under controlled conditions, such as reference face images taken with the mentioned multi-camera system using defined lighting, camera lenses, and parameters. The K13 database utilized in this study comprises a collection of face data gathered from 182 individuals who agreed to participate in the evaluation. A total size of 352 datasets, each consisting of 13 images captured from defined camera angles have been collected in the span of nine months. Of the 4.576 images, seven images have not been considered in the analysis, because the face recognition algorithm was not able to find the face from problematic angles, resulting in 4569 usable images.

Those images are not only used as a comparison for the evaluation of biometric systems and their algorithms, but also for the generation of semi-synthetic avatars for selected individuals. Using avatars is our initial approach towards a quality metric for synthetic face data. The basic idea of the first step is to produce avatar images as close to the real test subject images as the generating processes allow and then compare their performance in a 1:N analysis within a real database against the real images of the corresponding subjects. The similarity of the comparison scores, obtained using the same algorithms as mentioned in Section 3.1, are an indication of the realism and biometric comparability of these synthetic data.

### 4.2. Results

**Figure 3:**
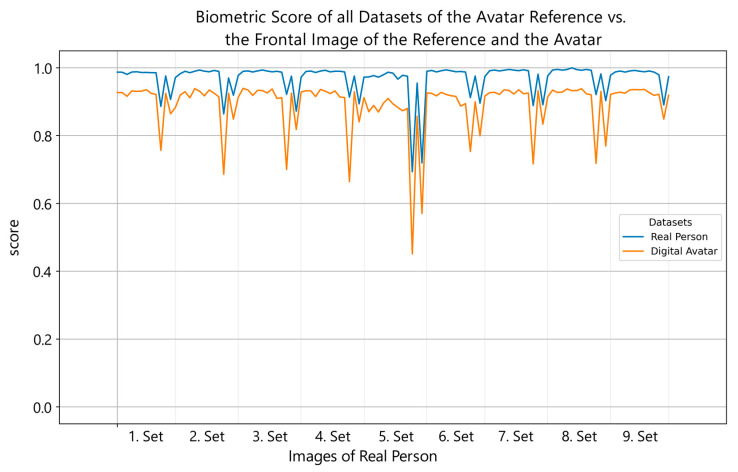
Line plot showing the performance scores of the frontal reference image (blue) and the frontal digital avatar image (orange) on the *y*-axis in comparison with all datasets from all perspectives of the reference individual, represented on the *x*-axis. The scores of the frontal avatar image have similar peaks when matched against the same candidate image as the reference. The peak intensities of the reference image are lower (about 50%) than the respective peaks of the avatar. The plots have an offset of about 0.06 at their baseline, the avatar image scoring lower, indicating that the avatar’s quality is not yet equal to its reference.

So far, three avatar datasets with 13 images each of digital representations were created and matched against all previously recorded authentic datasets of the real test subjects. Figure 3 shows the comparison of the frontal image of both the digital avatar and the reference, with nine real datasets for the same reference individual. As shown, synthetic data adapt to the authentic values and behave concurrently when compared to the reference dataset against the real reference data. The comparison score lines exhibit a certain degree of symmetry, but with a noticeable offset. As mentioned, initial versions of digital avatars achieved scores between 0.5 and 0.8. Currently, we are achieving a base offset of about 0.06 points. The performance drops when comparing with an upward or downward angled face, creating the pattern in Figure 3. A larger evaluation could show possible biases and more insights in our avatar datasets. A bias which needs further observation is a better-matching performance of older male avatars with their reference. Those tend to have imperfect skin and a more detailed face structure, which offers more information for face recognition algorithms to work with, and they are more difficult to reproduce.

**Figure 4:**
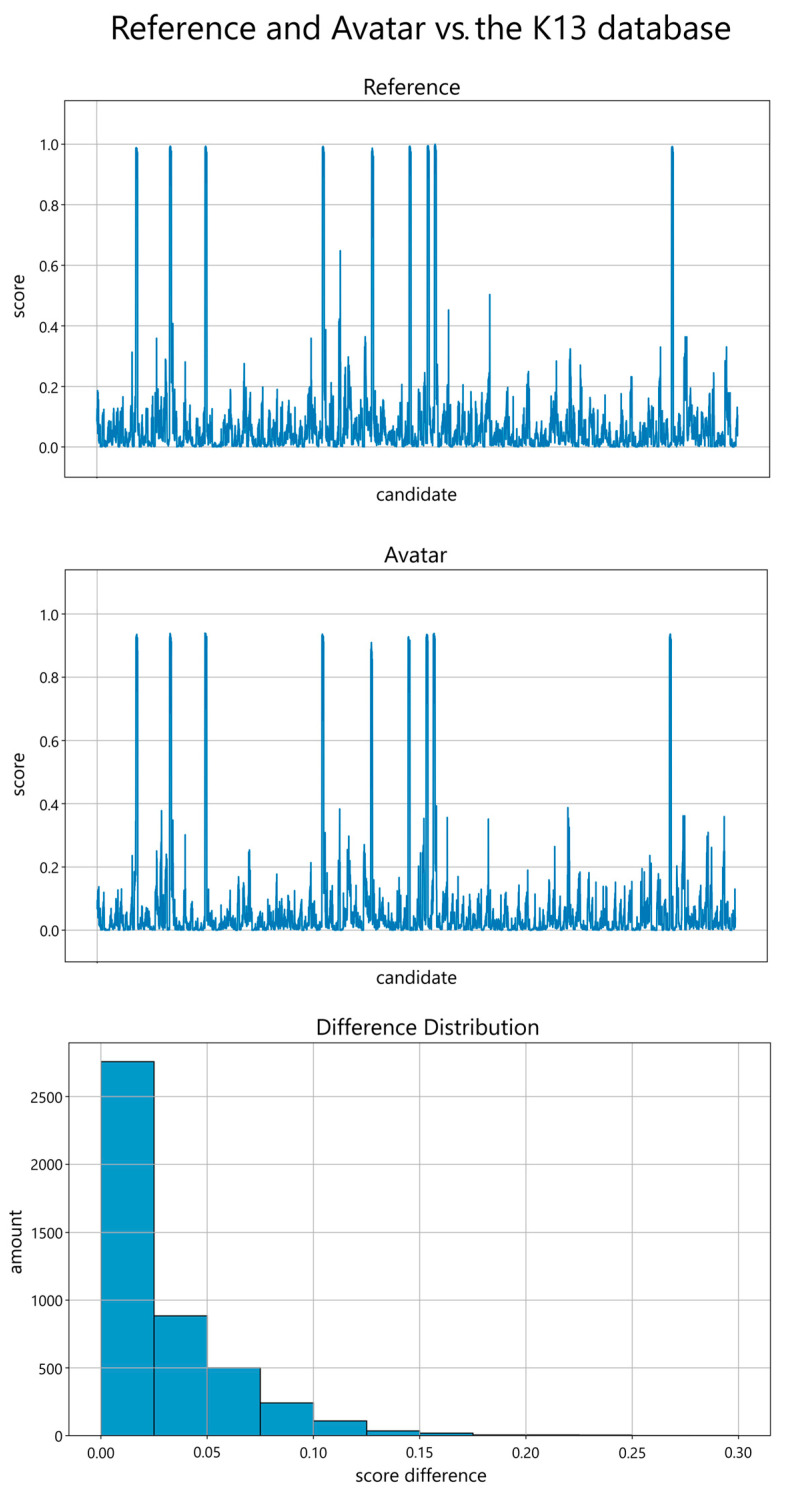
(**Top**, **Center**): Performance scores on the *y*-axis (ranging from 0.0 to 1.0 in steps of 0.2) of the frontal reference image (**top**) and frontal avatar image (**center**) in comparison with all K13 database images, including all 13 perspectives, as candidates on the *x*-axis. (**Bottom**): The distribution of the score differences grouped in 0.025-sized bins, ranging from 0.0 to 0.30, reveals a reduced disparity. The amount on the y-axis ranges from 0 to 2500 in steps of 500.

Figure 4 shows the comparison with the K13 database. The values do not exhibit symmetrical behavior when compared to the entire database. However, a pattern of correct decision direction can still be observed. Notably, when matched against the represented individual, a prominent spike occurs in the same location as the reference value’s spike, indicating a consistent alignment between the digital avatar’s performance and the reference dataset. It is also notable that even problematic angles for face recognition score higher than the scores of other individuals. The distribution of the data analyzed is 115 genuine and 4454 impostor samples. For the digital avatar, a score threshold of 0.5 results in a False-Match-Rate (FMR) of 0% and a False-Non-Match-Rate (FNMR) of 0.87%, with an accuracy of 99.57%, 100% precision, and a recall of 99.13%. A threshold of 0.8 results in FNMR of 9.57%, an accuracy of 95.22%, precision of 100%, and a recall of 90.4%. A higher threshold of 0.9 increases the FNMR to 26.09%, decreases the accuracy to 86.96%, and the recall to 73.92%, while the precision and FMR remain unchanged. This observation reinforces the avatar’s ability to accurately represent the individual it is based on, laying the foundation for further advancements in biometric research by paving the way for utilizing fully synthetic biometric data. A better recall could be obtained through further increasing the likeness of the digital avatars to their reference. With a decreased offset in Figure 3, it is likely that the comparison with the K13 database would be more symmetrical. Our K13 database consists primarily of students, faculty, and staff, which may not be representative of the broader population distribution. While universities often attract individuals from diverse cultural and ethnic backgrounds, the composition of the probands in our tests may not fully reflect this diversity. This could lead to an overrepresentation of certain characteristics and an underrepresentation of others. Impact analysis of different parameters, comparisons with larger databases, and other mesh creation techniques are part of future work.

## 5. Challenges and Limitations

While fully generated 3D characters are becoming increasingly realistic, it seems they are not yet capable of fully replacing real data in biometric research. For digital avatars, current comparison values fall below authentic values, indicating that digital avatars still have room for improvement in achieving accuracy and fidelity comparable to real data, but are already high enough to show a positive correlation and spot the reference person in a bigger database. Despite advancements in realism, digital avatars still fall under the acceptance threshold of human-like appearance. Although they appear almost human, they fail to capture the intricate details and nuances present in real individuals. To create digital avatars that accurately represent individuals with varying ethnicities, age groups, or physical attributes, more research is necessary to identify the corresponding parameter intervals. The unavailability of APIs or the inability to efficiently parse the user interface limits the tools which can be used to generate 3D data. Generating high-quality 3D characters is resource-intensive and rendering images may take a long time depending on the hardware available. It is prohibited to use MetaHuman and Reallusion Characters to train neural networks, further limiting their use.

## 6. Conclusions and Prospects

While the synthetic data generated in our example are not yet completely equal to real data, the results and current trend show a lot of promise. Simulating a real person as an avatar currently requires several hours of manual labor to generate promising results. Their matching results are highly dependent on the quality and visibility of the face details, such as texture, skin tone, and pores. Elevated results have been accomplished by improving avatar versions iteratively and upgrading their underlying maps for advanced visuals. Despite the positive results, creating and analyzing a larger and more diverse database of digital avatars would be crucial to ensure these are founded. Our work shows that the creation of synthetic data via character creation has not yet reached its full potential. The biggest challenge is a proper tool, which features reproducibility and automation, while providing high fidelity. Different tools potentially offer the desired features and are currently tested and will be investigated in future work.

With advancements in technology and the ever-growing realism in video game engines, further improvements in realism and detail are to be expected. Manually fine-tuning parameters for data generation can already yield acceptable results, and access to APIs would allow for the generation of arbitrarily large datasets. Unfortunately, APIs are not necessarily available, thus not all available tools can be used to generate large datasets without limits. As shown, both full and semi-synthetic characters generated with the presented tools already achieve results that lead to comparable outcomes when using face recognition algorithms. With further increased realism, it is expected that trained models can be implemented on synthetic-only databases, providing a better experience in biometric authentication scenarios. Our current method relies on the link between real data and fully synthetic data, digital avatars, which require real data to generate and analyze the performance. We are working towards making the leap and using only fully synthetic characters, while maintaining the high fidelity of digital avatars. Synthetic characters currently represent the face modality, but future works can expand their capabilities to include additional modalities, such as fingerprints and irises. This expansion would enable a more comprehensive and multi-modal approach.

Advancements in the field of synthetic biometric data benefit from the development of privacy-preserving biometric systems. By generating synthetic biometric data that retain the statistical characteristics and discriminatory properties of real data, but do not reveal sensitive information, privacy concerns can be mitigated. This allows for the development of biometric algorithms and systems without directly handling real individuals’ biometric information.

Synthetic data offer a controlled and scalable environment for algorithm development and testing, enabling researchers and developers to explore diverse scenarios and variations without relying solely on real data. By leveraging synthetic data alongside real data, biometric algorithms can be optimized and made more efficient. This approach helps models generalize well to real-world scenarios, bridging the domain gap and improving overall performance.

In addition to algorithm development, synthesis can be instrumental in creating benchmark datasets for evaluating and comparing biometric algorithms and systems. These benchmarks can provide standardized testing protocols, allowing fair comparisons between different approaches and facilitating advancements in the field. Another option is the training of a DNN for generating the parameters needed to produce digital avatars by providing synthetic characters and their parameter values as training data.

These prospects highlight the potential of synthetic data to address various challenges, improve biometric systems and their resilience, and drive advancements in the field. However, continued research, development, and validation are necessary to ensure that synthetic data accurately reflect real-world biometric characteristics and maintain their effectiveness in practical applications. 

## Data Availability

The study’s intention is to generate an open database with fully annotated synthetic characters for the development and evaluation of face biometric systems. We are currently working on a first part of the dataset that can be published.
